# The Effects of Different Attachment Types and Positions on Rotation Movement in Clear Aligner Treatments: A Finite Element Analysis

**DOI:** 10.7759/cureus.66273

**Published:** 2024-08-06

**Authors:** Erkan Sultanoğlu, Hakan Gürcan Gürel, Muzaffer Gülyurt

**Affiliations:** 1 Orthodontics and Dentofacial Orthopedics, Biruni University, Istanbul, TUR

**Keywords:** finite element analysis, rotation, mandibular premolar, attachment, clear aligner

## Abstract

Aim

Rotation of the mandibular premolars during aligner treatment is a difficult movement to achieve accurately. The purpose of this study is to compare the effects of different attachment types and positions used in clear aligner treatments on the rotation movement and retention of clear aligners in the rotated first premolar teeth. The study also addressed the stress values in periodontal ligaments (PDLs) with finite element analysis.

Materials and methods

For purposes of this research, we created a mandibular tooth model and modeled the premolar tooth with a 30° rotation. Twelve separate groups were created by attaching horizontal rectangular, vertical rectangular, ellipsoid, and semi-ellipsoid attachments to the premolar tooth in buccal, lingual, and combined buccal and lingual ways. A model without attachments was created to be used as the control group. An activation movement of 0.25 mm was applied to the first premolar tooth in all 12 models. The study evaluated clear aligner displacement, von Misses stress on the PDL, and tooth displacements using the finite element stress analysis method.

Results

It was found that the group with horizontal rectangular attachments placed on both the buccal and lingual sides had the highest stress value in the PDL (0.1971 MPa) and the highest displacement in the tooth (0.1267 mm). Conversely, the group with semi-ellipsoid attachments placed both buccally and lingually had the least displacement movement in clear aligners (0.1441 mm).

Conclusion

The results indicate that groups with attachments provided better retention than groups without attachments. Models with horizontal, rectangular attachments showed significantly more tooth displacement compared to other models. Horizontal rectangular attachments placed buccally and lingually combined to provide tooth movement in rotated mandibular first premolars can be recommended for clinical use.

## Introduction

To improve the control of tooth movements with clear aligners, researchers have attempted to regulate force transmission patterns and design new innovations [1]. To achieve complex movements, particularly rotations, composite attachments must be used on the teeth [2,3]. Attachments can increase the retention of clear aligners, control the direction of applied force, and enable specific tooth movements. In cases where extrusion, rotation correction, and root movements are required, attachments positioned automatically by computer-aided software are used. The recesses in the clear appliances, which create pressure points, are positioned in the areas of the root that require torque [4]. Attachments are created by applying a composite material to the teeth, using guide clear aligners to create spaces at the beginning of the treatment. Attachments are typically classified into three types: rectangular, beveled, and ellipsoid [5].

The rectangular attachment has a width of 2 mm, a height of 3-4-5 mm, and a thickness of 0.5-1 mm. When placed horizontally on the tooth, it can cause vertical movement. This type of attachment is preferred for tooth extrusion movement. Structurally, the attachment prevents slippage between the plaque and the tooth. When placed vertically on the tooth surface, it facilitates mesiodistal movements and makes it particularly beneficial for closing long gaps. The beveled attachment has a width of 3-4-5 mm, a height of 2 mm, and a thickness of 0.25-1.25 mm. Inclined attachments are necessary for tooth extrusion. Ellipsoid attachments measure 3 mm in height, 2 mm in width, and 0.75-1 mm in thickness. Incisors are used in pairs to rotate canines and premolar teeth or for root movement. When used independently, they should provide control over rotation [6]. However, research on aligners is limited, and further evaluation of their scientific properties is necessary [7].

To achieve rotational movement in teeth, it is essential to create space between them through expansion and interproximal reduction. Attachments designed for this purpose are recommended, especially for the round surfaces of canine and premolar teeth. Rotating round teeth is particularly challenging and requires careful consideration [8,9].

Finite element analysis (FEA) is a reliable and convenient method for evaluating stress around bones and implants. This analysis provides baseline data for new clinical methods and determines their potential effects. FEA generates computational data that reveals the behavior of new materials or techniques under simulated clinical conditions [10]. To the authors’ knowledge, no previous FEA study has evaluated the effects of attachment types and positions in clear aligner treatments for correcting mandibular first premolar tooth rotations. The null hypothesis was that attachment types and positions have no effect on tooth movement, clear aligner retention, or stresses in the periodontal ligament (PDL).

## Materials and methods

The mandibular bone model used in the study was created by modeling cortical bone, trabecular bone, teeth, and PDL. A 25-year-old male individual’s tomography was taken with a section thickness of 0.1 mm, and the resulting data was reconstructed. The individual’s mandibular bones and teeth were healthy. This reconstructed data was then transferred to 3D Slicer software in DICOM (.dcm) format. The computed tomography data in DICOM format was segmented in 3D Slicer software based on appropriate Hounsfield values and converted into a three-dimensional model. The resulting model was exported in STL format and imported into the Ansys SpaceClaim software, where a model of the mandibular bone was created.

Similar to FEA studies [9] in the literature, to create a 2 mm thick cortical bone model, a 2 mm offset was applied to the mandibular bone model. Trabecular bones were obtained by referencing the inner surfaces of the three-dimensional cortical bone with an adjusted thickness. PDL that was 0.25 mm thick was modeled using the outer surface of the teeth as a reference. The prepared models were placed in the correct coordinates in 3D space using Ansys SpaceClaim software, and the modeling process was completed.

Modeling attachments and clear aligners and creating working models

**Figure 1:**
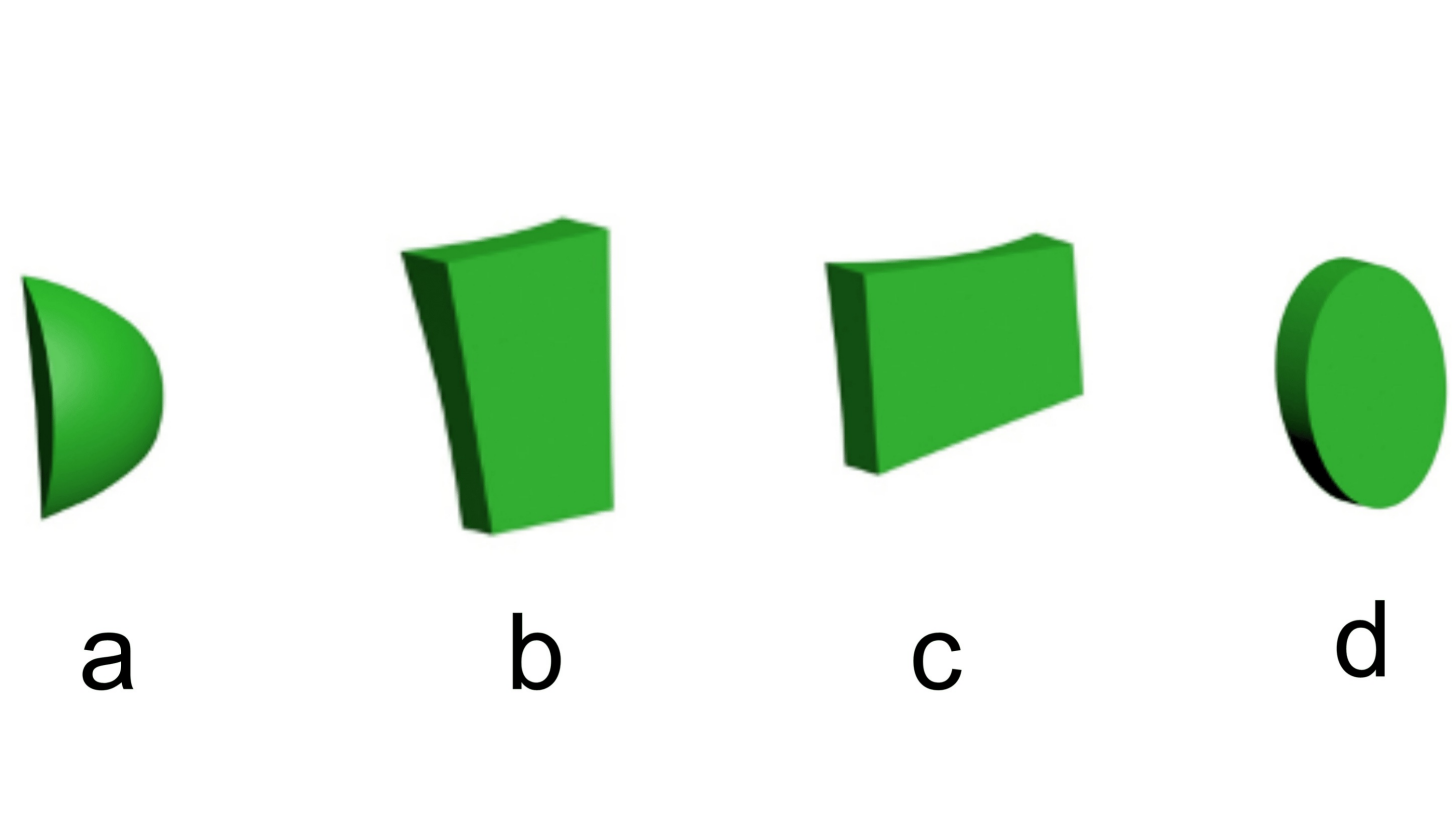
(a-d) Attachment shapes compared in the study (a) Semi-ellipsoid attachment. (b) Vertical rectangular attachment. (c) Horizontal rectangular attachment. (d) Ellipsoid attachment.

Attachments included a semi-ellipsoid attachment with a width of 2 mm, a height of 3 mm, and a thickness of 1 mm at the center; a vertical rectangular attachment with a width of 2 mm, a height of 3 mm, and a thickness of 1 mm; a horizontal rectangular attachment with a width of 3 mm, a height of 2 mm, and a thickness of 1 mm; and an ellipsoid attachment with a width of 2 mm, a height of 3 mm, and a thickness of 1 mm. The study employed four different attachments, each with specific dimensions and shapes, modeled using Ansys SpaceClaim software (Figure 1a-1d, Table 1).

**Table 1:** Attachment shape and placement of the groups compared in the study

Group	Attachment shape	Attachment placement
Group 01 (control group)		-
Group 02	Semi ellipsoid	Buccal
Group 03	Vertical rectangular	Buccal
Group 04	Horizontal rectangular	Buccal
Group 05	Ellipsoid	Buccal
Group 06	Semi ellipsoid	Lingual
Group 07	Vertical rectangular	Lingual
Group 08	Horizontal rectangular	Lingual
Group 09	Ellipsoid	Lingual
Group 10	Semi ellipsoid	Buccal + lingual
Group 11	Vertical rectangular	Buccal + lingual
Group 12	Horizontal rectangular	Buccal + lingual
Group 13	Ellipsoid	Buccal + lingual

**Figure 2:**
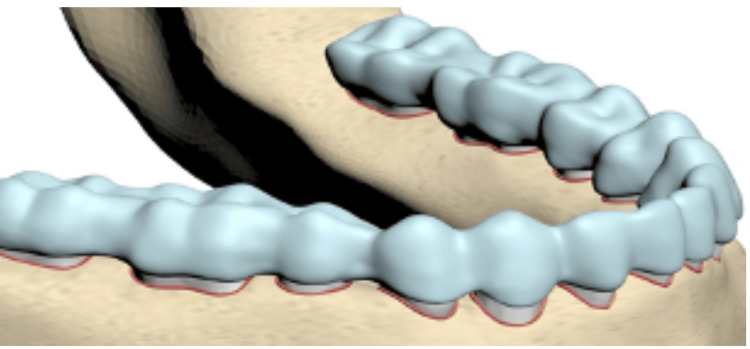
Picture of the model with the clear aligner used in the study

After repositioning the attachments, which were originally placed on the middle third of the mandibular first premolar tooth, both buccal and lingual 0.5 mm-thick clear plaques were modeled in Ansys SpaceClaim software to fit the attachments (Figure 2).

Obtaining Mathematical Models

Mathematical models were created by dividing geometric models into small, simple pieces called meshes. After completing the modeling process in Ansys SpaceClaim software, the models were mathematically created using Ansys Workbench software and prepared for analysis.

To perform the analyses, the mathematical models prepared in the Ansys Workbench software were transferred to the LS-DYNA solver. This program is primarily used for explicit solutions and also has an implicit solver that is also used for static problems.

Material Definitions

The analyzed model was defined numerically using linear material properties, including elastic modulus and Poisson’s ratio (Table 2).

**Table 2:** Physical properties of the substances modeled in the study PDL, periodontal ligament

Material	Elastic module (MPa)	Poisson’s ratio
Cortical bone	13,700	0.26
Trabecular bone	1,370	0.3
Dentin	18,600	0.3
PDL	0.667	0.45
Composite attachment	12,500	0.36
Aligner	528	0.36

Loading Scenarios and Boundary Conditions

In all models, a total activation force of 0.25 mm was applied to the buccal and lingual regions of the clear aligner on the first premolar tooth. Force was applied counterclockwise. This force represented a displacement of 0.125 mm from each surface. The models were fixed by restricting all degrees of freedom at the nodal points in the region of the bone, preventing movement in all three axes. The model includes all parts. The boundary condition was applied to ensure symmetry in the Y-Z plane, perpendicular to the X-axis. Thirteen nonlinear static analyses were performed on 13 models under specified force and boundary conditions.

Table 3 provides quantitative information for the 13 models created, including the combined systems and connection status between parts. To obtain accurate results when analyzing mathematical models, it is essential to define the surface relationships of the model’s parts in the analysis program. For all models, nonlinear friction contacts a coefficient of µ = 0.2 as defined on the aligner-tooth and aligner-attachment interfaces.

**Table 3:** Total element and node number of analysis models

Model	Total number of nodes	Total number of elements
Model 1	278,913	1,017,593
Model 2	280,915	1,024,468
Model 3	282,970	1,032,612
Model 4	283,295	1,033,275
Model 5	281,938	1,029,034
Model 6	279,784	1,020,124
Model 7	282,089	1,029,183
Model 8	283,016	1,032,448
Model 9	281,187	1,025,824
Model 10	284,293	1,036,566
Model 11	288,051	1,051,371
Model 12	285,580	1,040,676
Model 13	284,352	1,037,342

The study defines the bonded type of contact among other contacting components. This approach assumes that the parts operate with full correlation during their movement.

## Results

**Figure 3:**
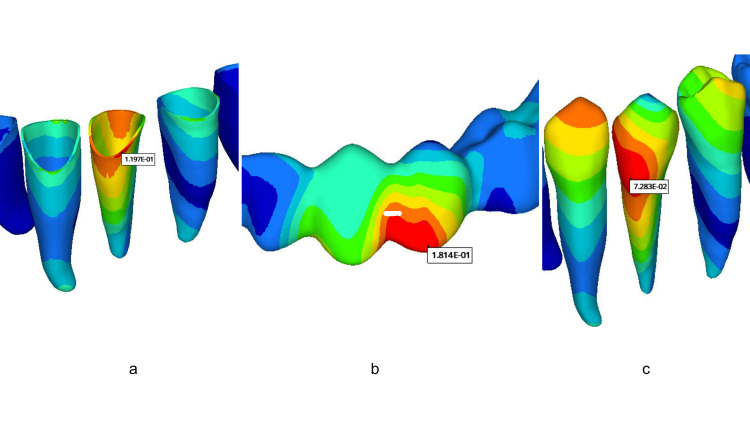
Example representations of model elements in color maps (a) Rotated premolar. (b) Clear aligner. (c) PDL. PDL, periodontal ligament

**Figure 4:**
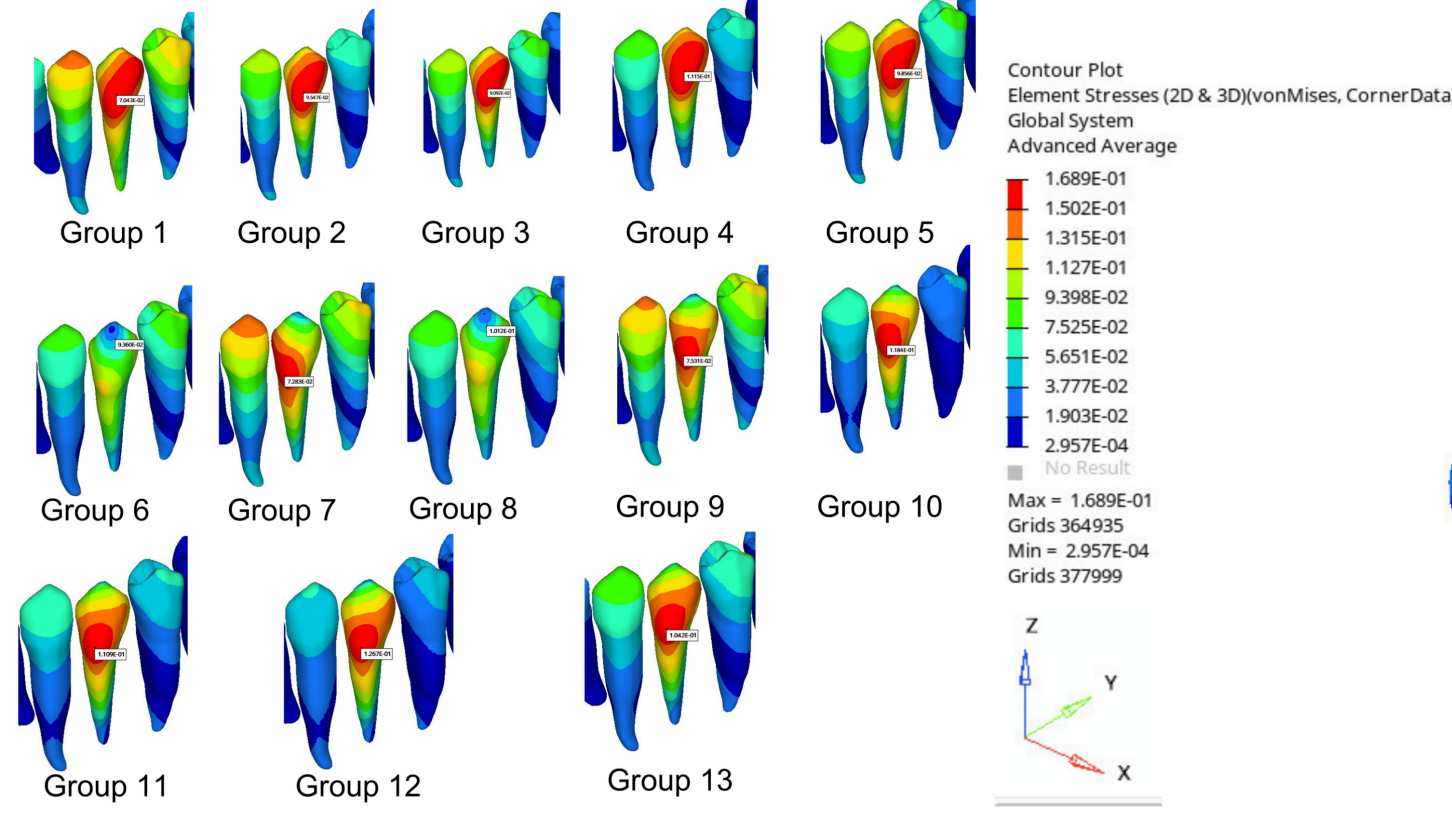
Displacement distance of a rotated premolar

**Figure 5:**
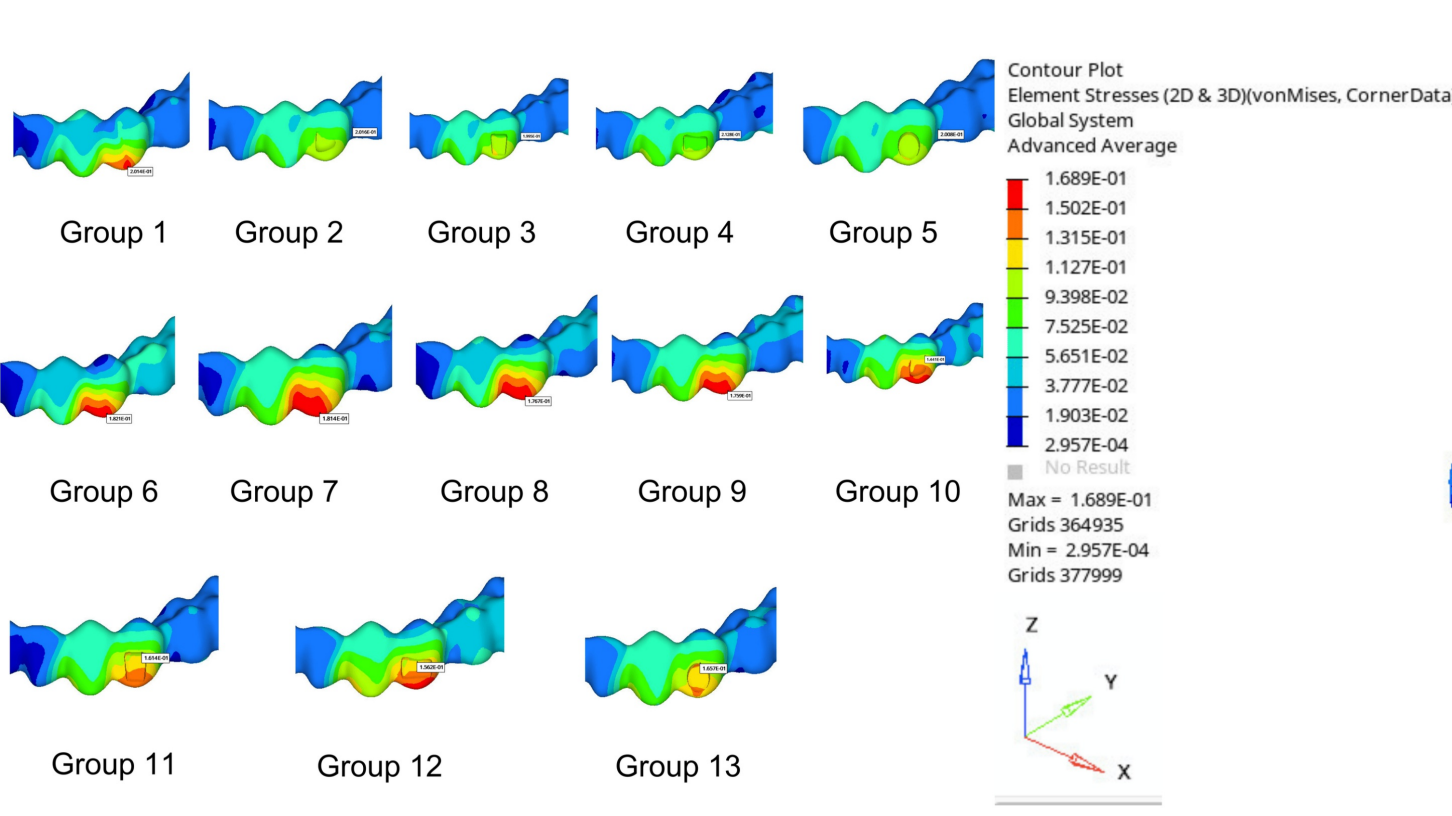
Displacement distances of clear aligners

**Figure 6:**
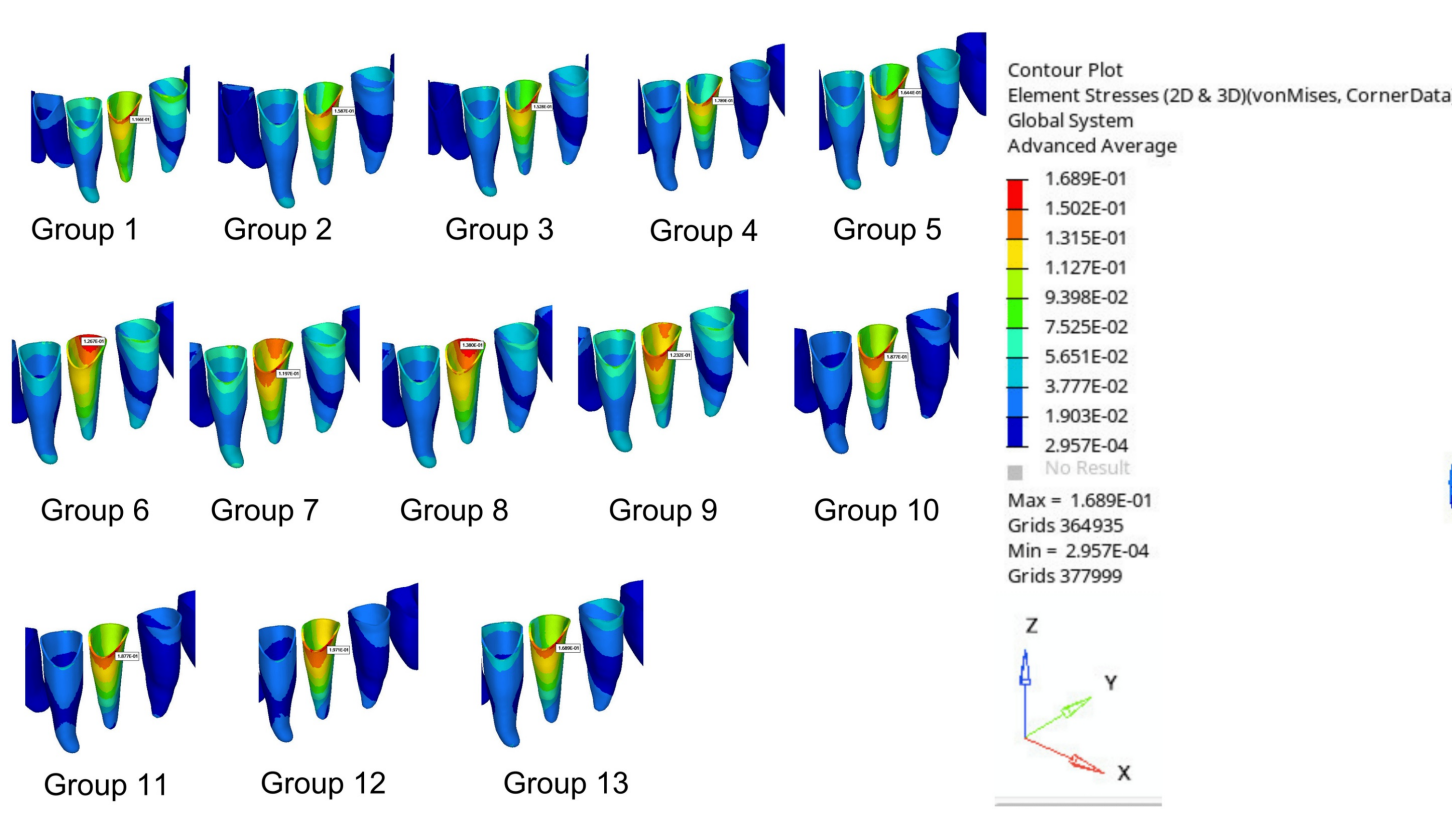
von Mises stress values on the PDL PDL, periodontal ligament

Table 4 shows tooth displacements, clear aligner displacements, and von Mises stress values occurring in PDL for the groups compared in the study. The von Mises stress distribution and the values of transversal, sagittal, and vertical displacement distributions of the selected nodes on the created models were obtained. The X-axis indicates the direction of the transverse displacement, the Y-axis indicates the direction of the sagittal displacement, and the Z-axis indicates the direction of the vertical displacement. The displacements and stresses obtained in the analysis are represented visually on a color scale within specified limits. The regions exhibiting the highest von Mises stress levels and movement are indicated in red, while those exhibiting the lowest levels are indicated in blue. The images of displacement amounts are evaluated separately in the sagittal, transversal, and vertical directions. The red areas indicate displacements in the direction of the specified axis, the blue areas indicate displacements in the opposite direction of the specified axis, and the green or yellow areas indicate the lowest amount of displacement in the direction of the specified axis. In this study, displacement amounts were expressed in millimeters (mm), and stress values were expressed in newtons per square meter (MPa) (Figures 3-6).

**Table 4:** Tooth total displacement, aligner total displacement, and von Mises stress values occurring in PDL for the groups compared in the study PDL, periodontal ligament

Group	(1) PDL von Mises stress (MPa)	(2) Aligner total displacement (mm)	(3) Tooth total displacement (mm)
Group 01 (control group)	0.1166	0.2014	0.07043
Group 02	0.1587	0.2016	0.09547
Group 03	0.1528	0.1995	0.09092
Group 04	0.1789	0.2128	0.1115
Group 05	0.1644	0.2008	0.09856
Group 06	0.1267	0.1821	0.0936
Group 07	0.1197	0.1814	0.07283
Group 08	0.138	0.1767	0.1012
Group 09	0.1232	0.1759	0.07531
Group 10	0.1877	0.1441	0.1184
Group 11	0.1749	0.1614	0.1109
Group 12	0.1971	0.1562	0.1267
Group 13	0.1689	0.1657	0.1042

The highest tooth displacement for the mandibular first premolar was observed in Group 12 (0.1267 mm), and the lowest was seen in Group 1 (0.07043 mm). The total tooth displacement is generally higher in models with buccal-lingual attachments than in other models: Group 10 (0.1184 mm), Group 11 (0.1109 mm), Group 12 (0.1267 mm), and Group 13 (0.1042 mm) (Figure 4). The displacement of the clear aligner was highest in Group 4 (0.2128 mm) and lowest in Group 12 (0.1562 mm). The aligner total displacement is higher in the control group and in general in models with buccal attachment placement than in other models: Group 1 (0.2014 mm), Group 2 (0.2016 mm), Group 3 (0.1995 mm), Group 4 (0.2128 mm), and Group 5 (0.2008 mm) (Figure 5). The highest von Misses stress in PDL was observed in Group 12 (0.1971 MPa), while the lowest was detected in Group 1 (0.1166 MPa). Stress values occurring in PDL were generally observed to be lower in models with lingual attachments than in other models. Group 6 (0.1267 MPa), Group 7 (0.1197 MPa), Group 8 (0.1380 MPa), and Group 9 (0.1232 MPa) (Figure 6).

## Discussion

The findings of the present study have led to the rejection of the null hypothesis. The study found that the shape and position of attachments used in clear aligner treatments have varying effects on tooth displacements, clear aligner displacements, and von Mises stresses in PDL. Although clear aligners are becoming more popular, there is often a discrepancy between the planned orthodontic movements in the virtual installation and the actual results [11]. Chisari et al. [12] reported that in clear aligner treatments, the movement of a single incisor tooth was 57% of the expected amount. In these treatments, movement was adjusted via software [13]. Auxiliary elements, such as power arms, elastics, and buttons, can also be used [14]. Factors such as the physical properties of the aligners and their production methods [13] and the position of the aligner relative to the gum [15] are not thought to significantly affect the outcome of the treatment due to the phasing of tooth movement.

The extant literature contains a variety of opinions regarding the degree of tooth movement activation in clear aligner treatments [16-21]. In a study, treatment plans were created in such a way that the activation amount in each plate would be 0.5 mm in clear aligner treatments, and it was reported that the stress on the teeth decreased rapidly. They explained the time-dependent stress reduction as the tooth moving away from the aligner [16]. Houle et al. [17]. suggested that a displacement amount of 0.25 mm per aligner should be applied in clear aligner treatments. Jiang et al. [18] indicated that the activation range for each aligner should be between 0.15 and 0.25 mm. They noted that an activation amount of 0.25 mm represents the maximum achievable tooth displacement, given the elasticity and thickness of the clear aligner materials. In this study, a total activation of 0.25 mm was applied from the active surfaces of the buccal and lingual regions of the clear aligner on the mandibular first premolar tooth in all models, resulting in a 0.125 mm displacement from each surface.

It was widely acknowledged that correcting rotational movement with clear aligners represents a particularly challenging task in the case of tapered teeth. Research indicates that clear aligner treatments without attachments result in less tooth displacement due to tipping forces on the teeth. In contrast, treatments with composite attachments increase the rotation force in clear aligner treatments [19,20]. The use of composite attachments in both vertical and horizontal shapes during clear aligner treatments has been shown to increase tooth displacement [21]. Studies have determined that clear aligners with ellipsoid and rectangular attachments result in more successful tooth displacement [16]. It has further been suggested that attachments can enhance the effectiveness of derotation movement and improve retention by creating undercuts for the clear aligner [20-22]. In this study, it was observed that tooth displacement was greater in models with attachment placement. It was also observed that lingual and buccal attachment designs, when used together, allowed greater tooth displacement than those attachments that were placed only lingually or buccally. The results suggest that the placement of attachments affects the retention of clear aligners.

Evaluation of the displacement of clear aligners

Movement values occurring in clear aligners are valuable for examining their retention. In our study, the highest displacements in clear aligners were observed in models with buccally placed attachments (Groups 2, 3, 4, and 5), and these values were close to the control group (0.2014 mm). Our study did not determine a clear order in the mobility of clear aligners based on attachment shapes. Since no similar study comparing attachment types was found in the literature, we could not discuss our results. Dasy et al. [7] evaluated the retention of clear aligners with ellipsoid and inclined rectangular attachments placed in the buccal area in their study. They further investigated the effects of aligner thicknesses and attachment shapes on clear aligner retention. According to the report [7], ellipsoid attachments did not have a significant effect on retention, whereas beveled attachments had a significant effect. It was observed that the group with the ellipsoid attachment had higher aligner displacement than the vertical rectangular attachment group and lower aligner displacement than the horizontal rectangular group when the attachment groups in the buccal area were examined. Unfortunately, we could not make a full comparison as we did not use an inclined attachment in our study.

Evaluation of von Misses stress distribution in the PDL

This research examined the stresses on PDLs and found that the models with buccal and lingual attachments had the highest stress values when the buccal-lingual combined attachment was used. The highest stress values for buccally and lingually placed attachments were observed in horizontal rectangular, ellipsoid, semi-ellipsoid, and vertical rectangular shapes, respectively. In models with buccal and lingual attachments, the order of attachment placement was a horizontal rectangle, semi-ellipsoid, vertical rectangle, and ellipsoid. The stress values in the control group without attachments were lower than in all models. Therefore, the model with the horizontal rectangular attachment placed in Group 12 (0.1971 MPa) had the highest stress value.

The model with a vertical rectangular attachment in Group 7 exhibited the lowest stress value (0.1197 MPa) compared to the other models. The observed stress values (0.1971 MPa and 0.1197 MPa) are within the range that can cause alteration of the PDL, leading to bone remodeling [23,24]. Cortana et al. [25] conducted a study to evaluate tooth displacements and deformation in the PDL of mandibular second premolars with different rotations, both with and without vertical rectangular attachments. They found that tooth displacement was significantly greater in groups with attachments compared to those without and that higher stresses occurred in the PDLs of teeth with attachments and higher rotation.

Evaluation of displacement numbers of teeth

The highest amounts were observed in the buccal-lingual, buccal, and lingual groups, respectively. In terms of rotation movement, models with all attachments provided more tooth displacement than the control group. The group with the highest total tooth displacement was Group 12, where the horizontal rectangular attachment was placed on the buccal and lingual surfaces (0.1267 mm). The study found that the attachment placed on the vertical rectangular lingual in Group 7 resulted in the lowest tooth displacement (0.07283 mm). When evaluating attachment shapes individually, buccally placed attachments resulted in greater tooth displacement compared to lingually placed designs. Savignano et al. [21] observed that ellipsoid attachments were more successful in achieving transversal and sagittal tooth displacements. In this study, we compared the displacement amounts of the model with an ellipsoid attachment and the model with a horizontal rectangular attachment. The results showed that the displacement amount of the model (Group 2) with ellipsoid attachment was lower. This difference can be attributed to the evaluation of different tooth displacements. Elkholy et al. [22] found that rotation movement was more successful in premolar teeth with ellipsoid attachments than in teeth without attachments. Similarly, our study determined that the first premolar tooth moved more in the model with ellipsoid attachment than in the model without attachment. Due to the limited number of studies evaluating the effect of attachment shapes and positions on the stresses on the teeth, aligner displacement, and PDL, we were unable to make a detailed comparison of our results.

Limitations of the study

FEA models generally assume linear, elastic, and homogeneous models. However, the fact that the mandibular bone structure is not homogeneous and has anisotropic properties causes different stress distributions and intensities. Additionally, this study is the product of a controlled virtual simulation and cannot describe situations where naturally varying and multidirectional stresses are present in the oral cavity [26-28].

## Conclusions

Considering the study’s limitations, it can be suggested that using attachments in clear aligner treatments positively influences tooth displacement and aligner retention. Evidence indicates that combining attachments on both buccal and lingual surfaces is more effective for rotating a tooth than using attachments on a single surface. However, placing attachments on two surfaces increased stress values in the PDL, although this did not exceed biological limits. Further advanced, comprehensive, and clinical studies are needed on this topic.
